# Supplementary material for: Bone marrow central memory and memory stem T-cell exhaustion in AML patients relapsing after HSCT
Source: Nat Commun. 2019 Mar 25;10:1065. doi: 10.1038/s41467-019-08871-1 (PMC6434052; doi:10.1038/s41467-019-08871-1)
Supplement: Supplementary file 1 — Supplementary Information [file 41467_2019_8871_MOESM1_ESM.pdf]

Bone marrow central memory and memory stem T-cell exhaustion in AML patients relapsing after HSCT

Noviello, Manfredi et al.

Supplementary Information

Supplementary figures

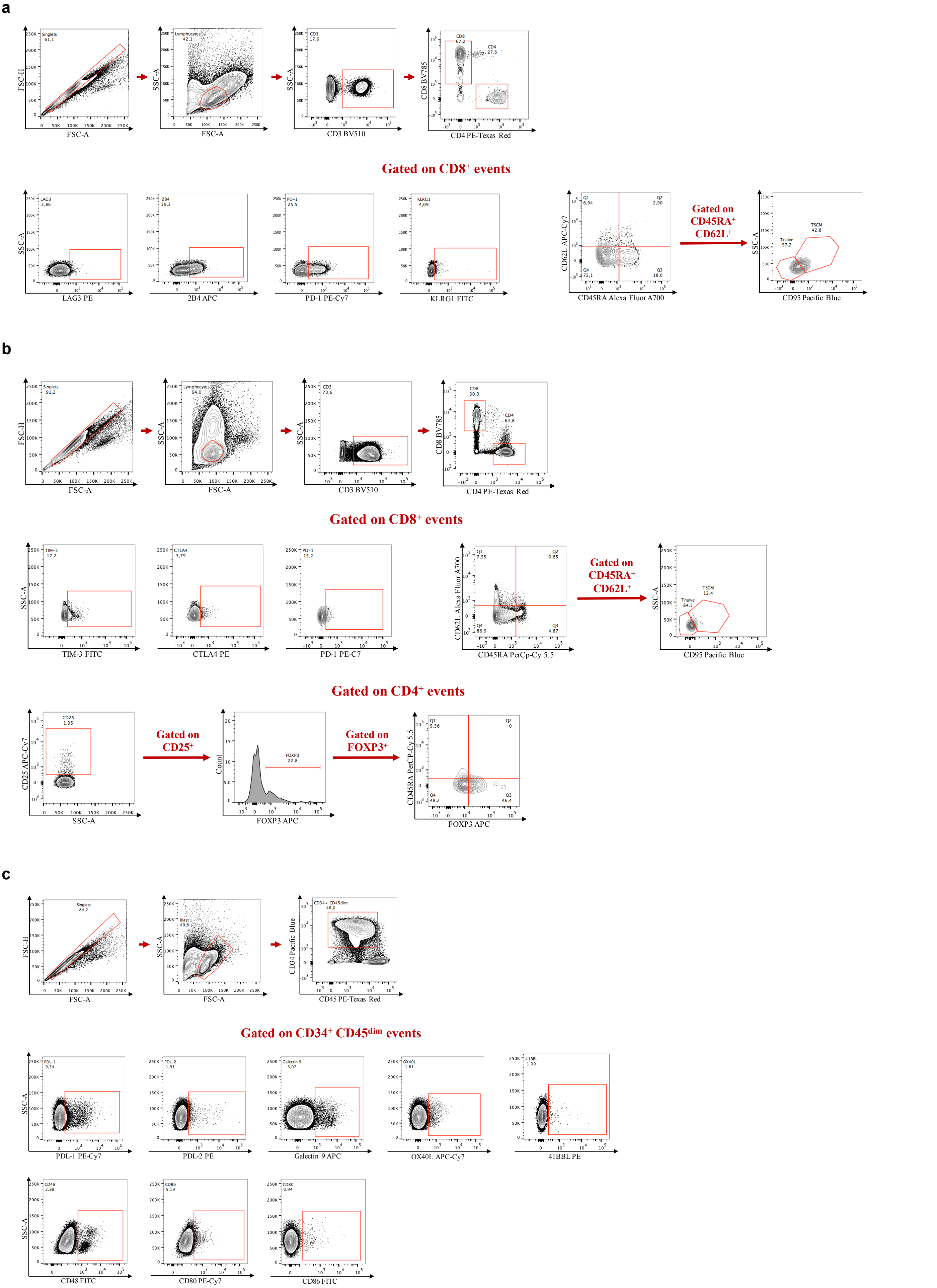

Supplementary Figure 1. *Gating strategy used for multiparametric flow cytometry analysis*. Sequential gating for the analysis reported in Fig.1, Fig.3 and Supplementary Fig.2, for all the different flow cytometry panels used. (a) Inhibitory receptors and memory differentiation markers on the cell surface. (b) Inhibitory receptors and Treg differentiation markers on the cell surface and after cell fixation and permeabilization. (c) Gating strategy for the analysis of blasts at relapse.

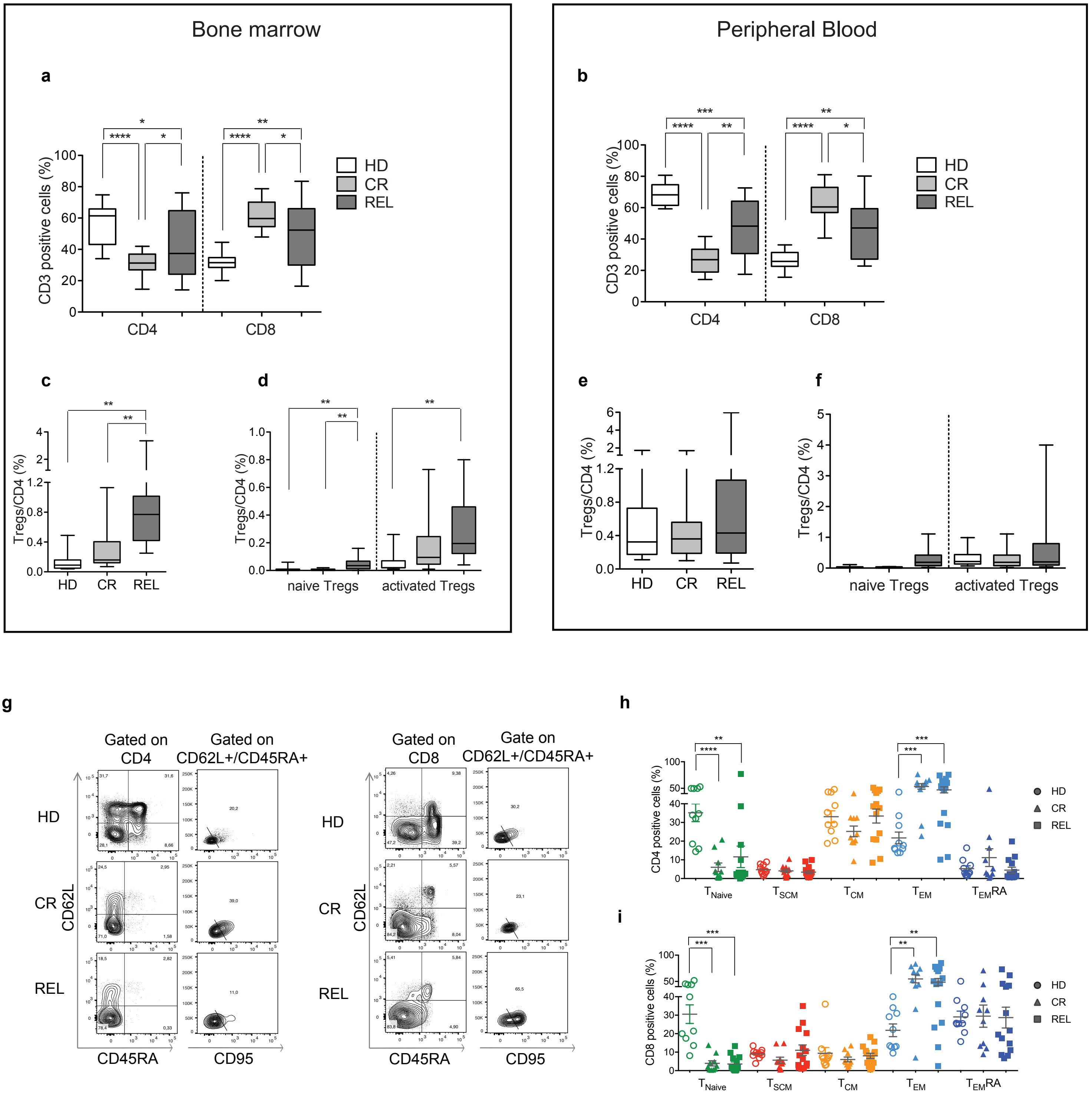

Supplementary Figure 2. *T-cell subset distribution in the bone marrow and in peripheral blood of HSCT patients according to transplant outcome.* The T-cell subset distribution of healthy donors (HD), patients who achieved long-term complete remission (CR) and patients who experienced relapse (REL) after HSCT was evaluated in the Bone Marrow (HD=10, CR=16, REL=16) and in Peripheral Blood (HD=10, CR=14, REL=10). (**a-b**) Proportion of $CD4^+$ and $CD8^+$ subsets on total $CD3^+$ T cells in HD, CR and REL patients for BM-infiltrating (**a**) and circulating (**b**) T cells. (**c-f**) Relative proportion of Tregs and proportion of naïve and activated Tregs over total $CD4^+$ T cells in either BM-infiltrating (**c-d**) and circulating (**e-f**) T cells. (**g**) Representative plots of the gating strategy for the visualization of T-cell memory subsets in one HD, one CR and one REL patient. (**h-i**) Relative proportion of naïve ($CD45RA^+CD62L^+CD95^-$, T_{Na}), memory stem ($CD45RA^+CD62L^+CD95^+$, T_{SCM}), central memory ($CD45RA^-CD62L^+$, T_{CM}), effector memory ($CD45RA^-CD62L^-$, T_{EM}) T cells and terminal effectors ($CD45RA^+CD62L^-$, $T_{EM}RA$) over the total $CD4^+$ (**h**) or $CD8^+$ (**i**) T-cell subsets in peripheral blood samples harvested from HD, CR or REL. Individual data points, means, and SEM are shown. Median, min and max values are reported for box-and-whiskers plots. *, $p<0.05$; **, $p<0.01$; ***, $p<0.001$; ****, $p<0.0001$, nonparametric unpaired two-sided T-test.

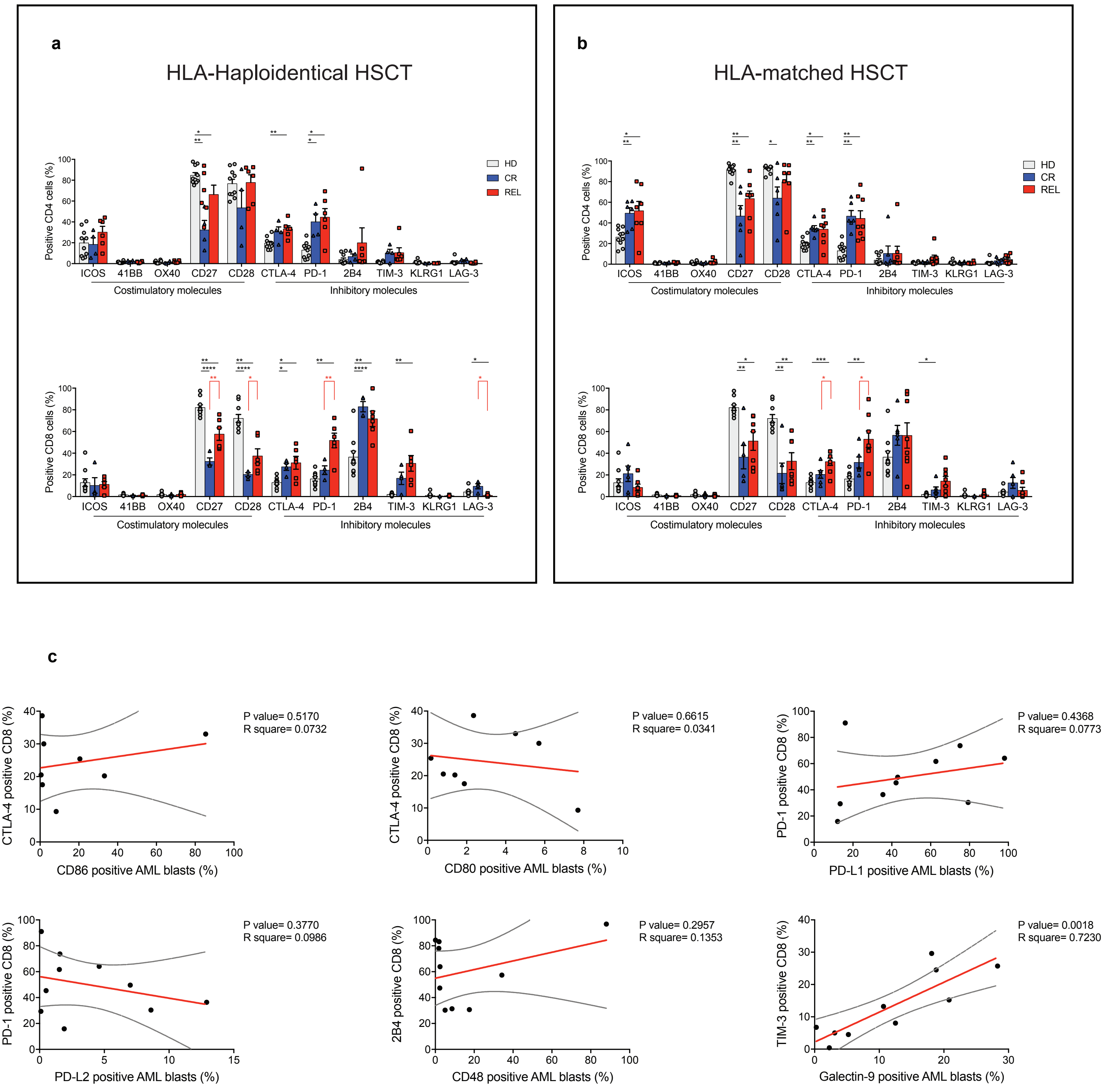

Supplementary Figure 3. *Expression of inhibitory receptors and costimulatory molecules on circulating T cells of HSCT patients according to clinical outcome and transplant type.* (**a**-**b**) HLA-matched (N=14) and HLA-haploidentical (N=10) patient samples were analyzed for the expression of inhibitory and costimulatory molecules on circulating T cells; peripheral blood samples from healthy donors were used as controls (HD, N=10). The percentage of $CD4^{+}$ and $CD8^{+}$ BM-T cells positive for costimulatory or inhibitory receptors in the HLA-haploidentical (**a**) and HLA-matched (**b**) transplant settings is reported (HD CR REL). Individual data points, means, and SEM are shown. Statistically significant differences between CR and REL groups are highlighted in red, the differences between patients' groups and HD in black. *, $p<0.05$; **, $p<0.01$; ***, $p<0.001$; ****, $p<0.0001$, nonparametric unpaired two-sided T-test. (**c**) Linear regression analysis between the relative proportion of PD-1, 2B4, CTLA-4 and TIM-3-expressing $CD8^{+}$ BM-T cells of patients undergoing HLA-identical HSCT and the relative proportion of their respective ligands on leukemic blasts. Linear regression, confidence intervals, P-value and R square are shown. All the inhibitory receptors have been visualized by means of cell surface staining apart for CTLA-4, visualized after cell fixation and permeabilization.

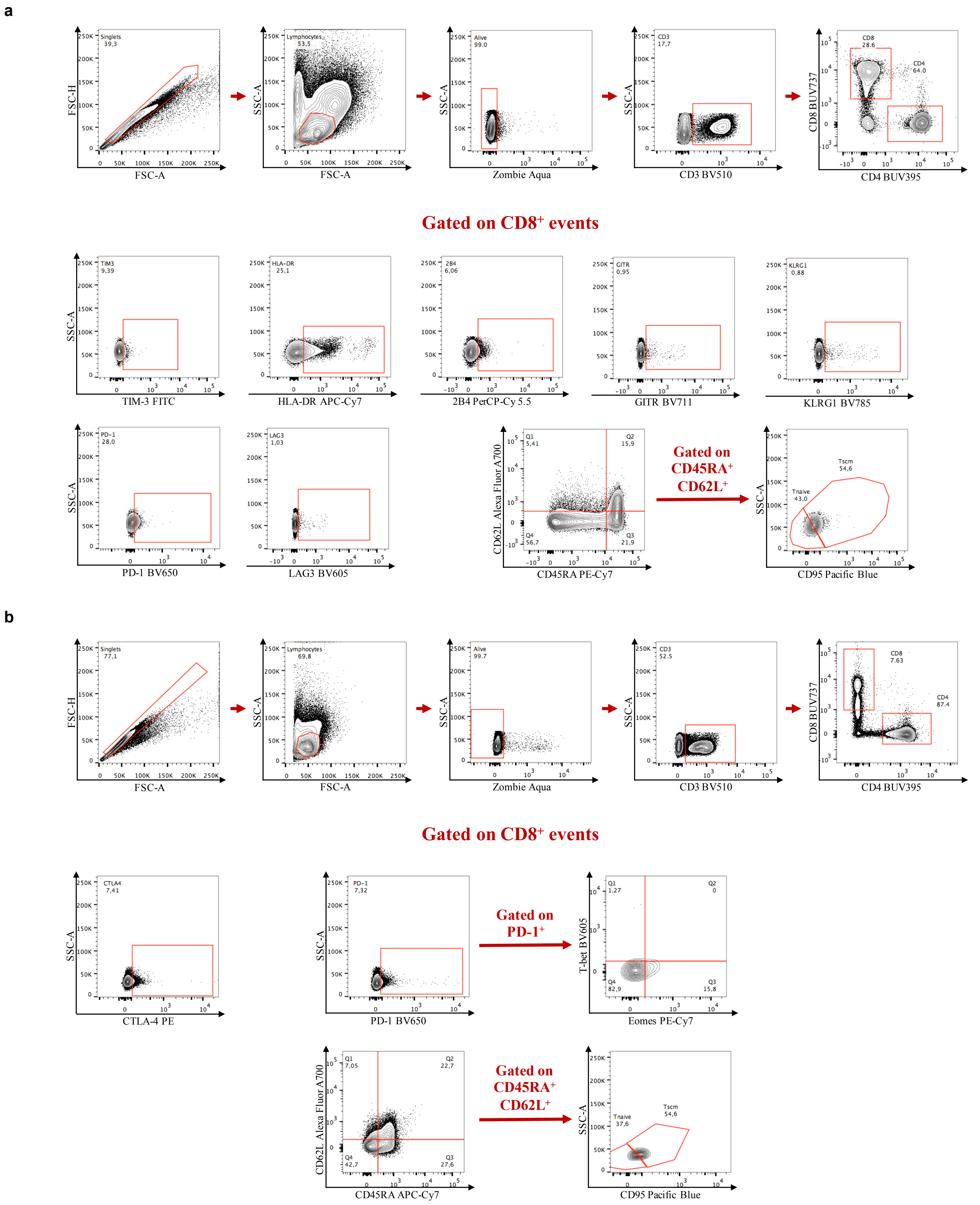

Supplementary Figure 4. *Gating strategy used for multiparametric flow cytometry analysis*. Sequential gating for the analysis reported in Fig.6 and Supplementary Fig.5, for all the different flow cytometry panels used. (a) Inhibitory receptors and memory differentiation markers on the cell surface. (b) Inhibitory receptors and transcription factors visualization on the cell surface and after cell fixation and permeabilization.

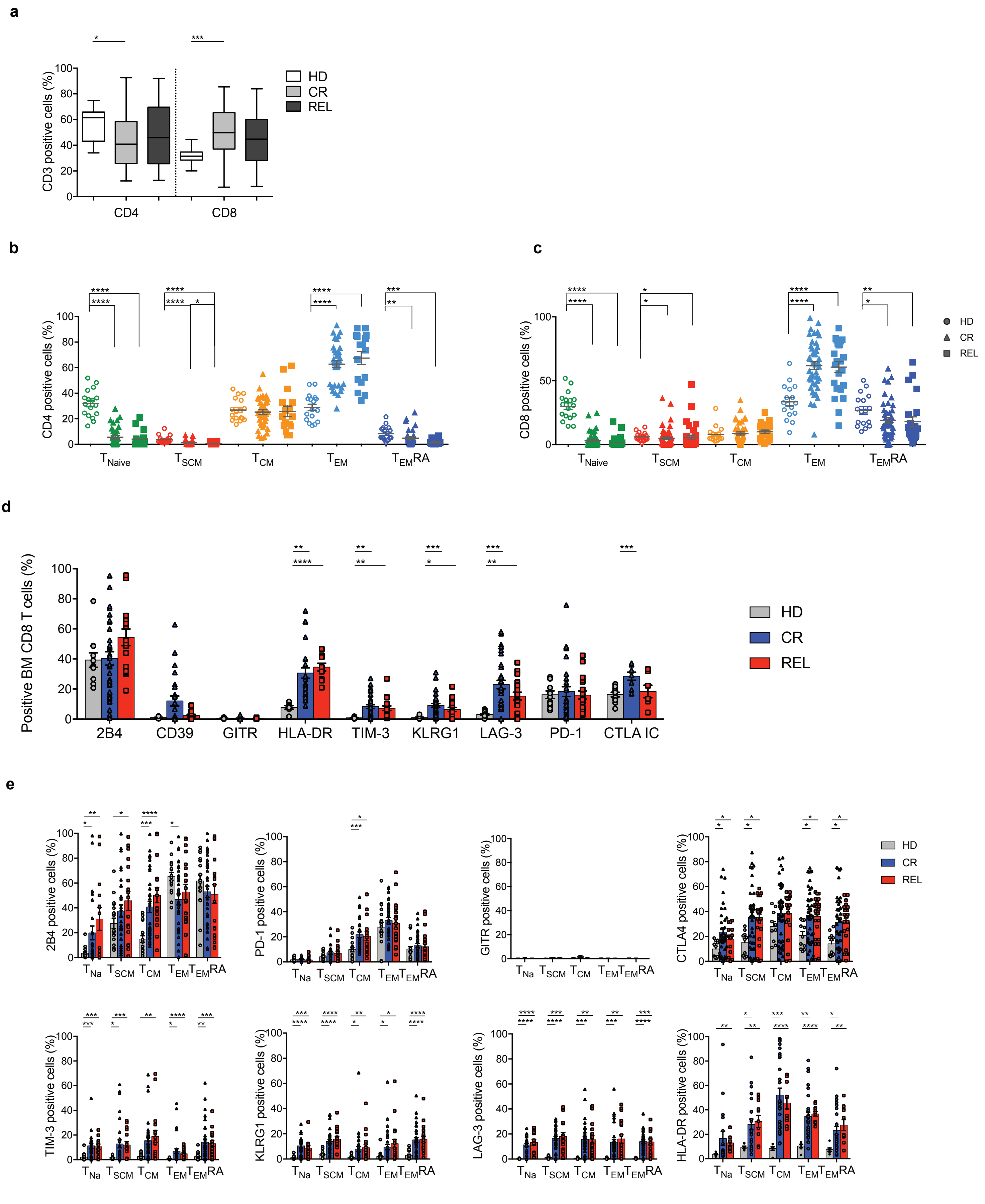

Supplementary Figure 5. *Circulating and BM-T cell subsets and exhaustion profile of CD8+ BM-T cells at early time points.* The T-cell subset distribution was evaluated in the Bone Marrow of HLA-matched transplanted patients who will maintain long-term complete remission (CR, N=37) or will experience disease relapse (REL, N=20) at the median time of 68 days after HSCT, when all patients were in CR. BM-T cells from healthy donors were used as controls (HD, N=18). (**a**) Proportion of $CD4^{+}$ and $CD8^{+}$ subsets on total $CD3^{+}$ BM-T cells in HD, CR and REL patients. (**b-c**) Relative proportion of memory T-cell subsets over the total $CD4^{+}$ (**b**) and $CD8^{+}$ (**c**) T cells, in each study group. (**d-e**) Percentages of BM-T cells expressing inhibitory receptors in both total $CD8^{+}$ T cells (**d**) and in each $CD8^{+}$ T-cell memory subset in each study group (**e**). All the inhibitory receptors have been visualized by means of cell surface staining apart for CTLA-4, visualized after cell fixation and permeabilization. Statistically significant differences between CR and REL groups are highlighted in red, the differences between patients' groups and HD in black. Individual data points, means, and SEM are shown. *, $p<0.05$; **, $p<0.01$; ***, $p<0.001$; ****, $p<0.0001$, nonparametric unpaired T-test.

Supplementary Table

Fluorochrome-conjugated monoclonal antibody	Clone	Vendor	Catalogue number	Dilution
2B4-APC	CI.7	BioLegend	329512	1:400
2B4-PerCP-Cy5.5	C1.7	Biolegend	329516	1:100
41BB-AF700	4B4-1	BioLegend	304120	1:70
41BBL-PE	5F4	BioLegend	311504	1:100
CD107a-FITC	H4A3	BD Biosciences	555800	1:50
CD117-BV510	104D2	BioLegend	313220	1:100
CD11b-BV785	ICRF44	BioLegend	301345	1:200
CD25-APC/Cy7	BC96	BioLegend	302614	1:100
CD27-FITC	M-T271	BD Pharmingen	555440	1:100
CD28-PE	L293	BD Pharmingen	555729	1:100
CD3-BV510	OKT3	BioLegend	317333	1:200
CD3-BV510	SK7	BioLegend	344828	1:200
CD33-AF700	WM-53	eBiosciences	56-0338	1:200
CD34-PB	581	BioLegend	343512	1:100
CD4-BUV395	SK3	BD Biosciences	563550	1:200
CD4-PE/Dazzle	RPA-T4	BioLegend	300548	1:200
CD45-PE/Dazzle	HI30	BioLegend	304052	1:200
CD45RA-AF700	HI100	BioLegend	304120	1:400
CD45RA-PeCy7	HI100	Biolegend	304126	1:100
CD45RA-PerCP/Cy5.5	HI100	BioLegend	304122	1:100
CD48-FITC	BJ40	BioLegend	336706	1:400
CD62L-AF700	DREG-56	BioLegend	304820	1:70
CD62L-APC/Cy7	DREG-56	BioLegend	304814	1:100
CD70-PerCp/Cy5.5	113-16	BioLegend	355108	1:200
CD8-BUV737	SK1	BD Biosciences	564629	1:200
CD8-BV785	RPA-T8	BioLegend	301046	1:200
CD80-Pe/Cy7	2D10	BioLegend	305218	1:200
CD86-FITC	FUN-1	BD Pharmingen	555657	1:100
CD95-PB	DX2	BioLegend	305619	1:70
CD95-PE/Cy7	DX2	BioLegend	305622	1:200
CTLA-4-PE	BNI-3	BD Pharmingen	560939	1:100
Eomes APC/Cy7	WD1928	Invitrogen	25-4877-42	1:70
FOXP-3-AF647	259D	BioLegend	320214	1:100
Galectin-9-APC	9M1-3	BioLegend	348908	1:70
GITR-BV711	108-17	Biolegend	371212	1:70
HLA-DR-AF700	L243	Biolegend	307626	1:100
ICOS-PB	C398.4A	BioLegend	313522	1:100
ICOSL-APC	136726	R&D	FAB165A	1:70
IFN- -APC/Cy7	4S.B3	BioLegend	502530	1:100
IL-2-PB	MQ1-17H12	BioLegend	500324	1:100
KLRG1-BV785	2F1-KLRG1	Biolegend	138429	1:70
KLRG1-FITC	2Fi/KLRG1	BioLegend	138410	1:100
LAG-3-PE	Leu23-Leu450	R&D	FAB2319P	1:70
LAG3-BV605	11C3C65	Biolegend	369324	1:70
OX40-APC	ACT35	BioLegend	350008	1:100
OX40L-Biotin	ACT35	BioLegend	326306	1:100
PD-1-BV650	EH12.2H7	Biolegend	329950	1:200
PD-1-PE/Cy7	EH12.2H7	BioLegend	329918	1:70
PD-L1-PE/Cy7	29E.2A3	BioLegend	329718	1:70
PD-L2-PE	24F.10C12	BioLegend	329606	1:200
T-bet-BV605	4B10	Biolegend	644817	1:70
TFN-α-PE/Cy7	Mab11	BioLegend	502930	1:100
TIM-3-AF488	295D	R&D	FAB2365G	1:70

Supplementary Table 1. *List of the used fluorochrome-conjugated Monoclonal Antibodies.*
